# Advances in anti-EV-A71 drug development research

**DOI:** 10.1016/j.jare.2023.03.007

**Published:** 2023-03-30

**Authors:** Shuqi Wang, Zehan Pang, Huahao Fan, Yigang Tong

**Affiliations:** a College of Life Science and Technology, Beijing University of Chemical Technology, Beijing, China; b Beijing Advanced Innovation Center for Soft Matter Science and Engineering, Beijing University of Chemical Technology, Beijing, China

**Keywords:** Enterovirus A71, Hand, foot and mouth disease, Drug therapy, Antivirals, Drug design and discovery

## Abstract

**Background:**

Enterovirus A71 (EV-A71) is capable of causing hand, foot and mouth disease (HFMD), which may lead to neurological sequelae and even death. As EV-A71 is resistant to environmental changes and mutates easily, there is still a lack of effective treatments or globally available vaccines.

**Aim of Review:**

For more than 50 years since the HFMD epidemic, related drug research has been conducted. Progress in this area can promote the further application of existing potential drugs and develop more efficient and safe antiviral drugs, and provide useful reference for protecting the younger generation and maintaining public health security.

**Key Scientific Concepts of Review:**

At present, researchers have identified hundreds of EV-A71 inhibitors based on screening repurposed drugs, targeted structural design, and rational modification of previously effective drugs as the main development strategies. This review systematically introduces the current potential drugs to inhibit EV-A71 infection, including viral inhibitors targeting key sites such as the viral capsid, RNA-dependent RNA polymerase (RdRp), 2C protein, internal ribosome entry site (IRES), 3C proteinase (3C^pro^), and 2A proteinase (2A^pro^), starting from each stage of the viral life cycle. Meanwhile, the progress of host-targeting antiviral drugs and their development are summarized in terms of regulating host immunity, inhibiting autophagy or apoptosis, and regulating the cellular redox environment. In addition, the current clinical methods for the prevention and treatment of HFMD are summarized and discussed with the aim of providing support and recommendations for the treatment of enterovirus infections including EV-A71.

## Highlights

• The structural characteristics of EV71 and the mechanism of infection were reviewed.
• Anti-EV71 drug candidates for different targets based on *in vitro*, animal, and clinical data were summarized.
• The main therapeutic approaches for EV71 infection were discussed, and suggestions were made for further drug development.

## Graphical abstract

**Figure:**
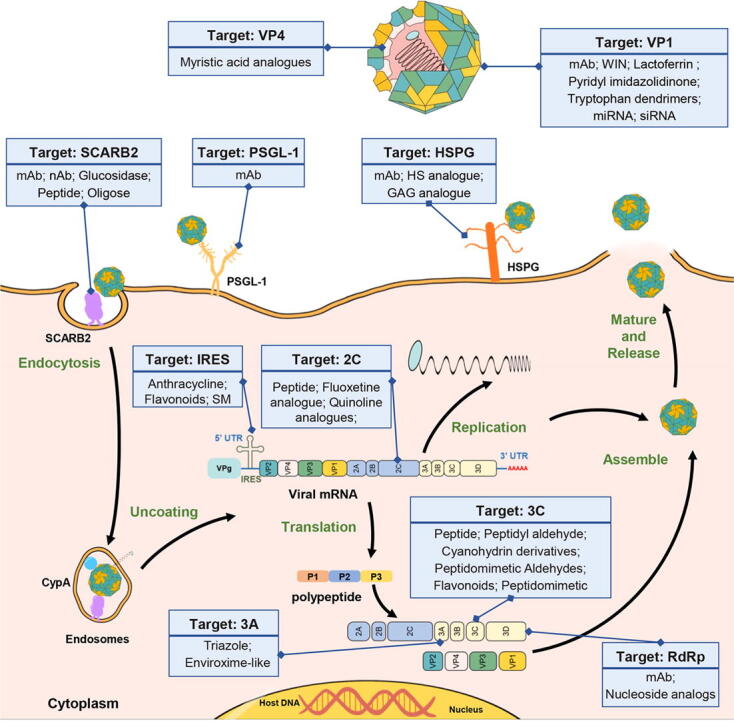

## Introduction

Enterovirus A71 (EV-A71) was first isolated in 1969 from the faeces of an infant with aseptic meningitis in the United States [1]. The virus has subsequently caused multiple pandemics in Bulgaria, Hungary, and many Asia-Pacific regions including Japan, Taiwan [2], Australia, Singapore, and Vietnam [3]. EV-A71 and Coxsackievirus (CV) A16 are major pathogens of hand, foot and mouth disease (HFMD), which is mainly transmitted by contact (faecal-oral transmission) and is prevalent in children under five years of age [4]. Most patients recover spontaneously within a short period, while a small number of patients develop a variety of complications including myocarditis, pulmonary edema, polio-like paralysis, aseptic meningitis, encephalitis, and other serious symptoms or even death.

Since 2008, HFMD (category C infectious disease) has steadily ranked first in the incidence of statutory infectious diseases in China . As one of the major pathogens of HFMD, the neurotropism of EV-A71 and the positive correlation between high EV-A71 load and clinical severity suggest that clinically available antiviral drugs should be developed as soon as possible [3]. Currently, there are no specific drugs or specific treatments for EV-A71 infection, but rather general routine antiviral and symptomatic management. As early as 2016, an inactivated EV-A71 C4 subtype vaccine with a clinical antiviral immune response of more than 98.8% was approved for marketing in China [5]. However, the existence of 11 different EV-A71 subtypes suggests that the virus is constantly mutating [6], and vaccine-induced antibodies tend to be specific; therefore, vaccine prevention combined with drug therapy can better control EV-A71 transmission and disease progression.

Since EV-A71 was first isolated, researchers worldwide have conducted drug development and clinical trials to seek various therapies against hand, foot and mouth disease and other complications caused by EV-A71 infection. Up to now, several drugs have entered the clinical validation stage, such as Acyclovir, Suramin (NCT03804749), Xiaoer Jiebiao Oral liquid in combination with Ribavirin (NCT02328651), Xiyanping injection (NCT01554930) [7], and other Chinese pharmaceutical preparations. Based on published articles and clinical data, this review sort out the anti-EV-A71 drugs, both in terms of targeting the virus and host. Table 1 provides a summary of the target, antiviral efficacy, resistant mutants, and current study status of these antiviral drugs against enterovirus A71 infection.

**Table 1:** List of antivirals against EV-A71 infection tested *in vitro* and *in vivo.*

**Drug action stage**	**Antivirals**	**Classes**	**Target**	**EV-A71 genotype tested**	**IC_50_/EC_50_**	**CC_50_**	**Resistant mutants**	**Current status (reference)**
Viral attachment and entry	JL2	mAb	SCARB2	C4	2 μg/mL (P < 0.01 by Student’s *t*-test)			*In vitro*[28]
	A9	NAb	SCARB2, capsid	C4	neut_50_ = 0.1 nM			*In vitro*[29]
	D6	NAb	SCARB2	C4	neut_50_ = 1 nM			*In vitro*[29]
	Cerezyme (contain GBA1)	Glucosidase	SCARB2	ME40, ME42, ME71, ME72 (Isolate from Japan)	100 μg/mL (P < 0.001 by Student’s two-sided *t*-test)			*In vitro*[219]
	SP40	Peptide	SCARB2	A, B3, B4, C2	6–9.3 µM	>280 μM		*In vitro*[30]
	Acarbose	Oligose	SCARB2	B2 (GFP-EV-A71)	1.5 μg/mL (P < 0.001)			*In vitro,* *In vivo*: ICR mice[31]
	KPL1	mAb	PSGL-1	C2, C4	10 μg/mL (P < 0.01 by Student’s two-tailed *t*-test)			*In vitro*[220] *In vitro*[32]
	Hep	HS analogue	HS	C2	205.00 μg/mL	48430.2 μg/mL		*In vitro*[34] *In vitro*[35]
	HS	HS analogue	HS	C2	290.00 μg/mL	205.00 μg/mL		*In vitro*[34]
	PPS	HS analogue	HS	C2	238.00 μg/mL	10795.47 μg/mL		*In vitro*[34]
	9 (NG), 22 (NG-like), 13 (NI), 21 (NI-like), 23 (NI-like)	HS analogue	HS	C2	7.9–30 μM	ND or >1000 μM		*In vitro*[33]
	Fondaparinux	GAG analogue	HS	C2	9.4 μM	ND		*In vitro*[33]
	D5	mAb	SCARB2, PSGL-1, HS, VP1	C4	0.203 μg/mL		VP1: K218E (3/9), K218T (6/9)	*In vitro*[36]
	C4	mAb	SCARB2, PSGL-1, HS, VP1	C4	0.952 μg/mL		VP1: K218E (4/9), K218T (2/9), K218N (3/9)	*In vitro*[36]
	H7	mAb	VP1	C4	0.287 μg/mL			*In vitro*[36]
	Pleconaril	WIN	VP1	A	0.13–0.54 μg/mL	12.5– 25 μM	C4	*In vitro*, *In vivo*: Neonatal ICR mice[43], Clinical: NCT00031512[10], [42]
	BPROZ-194	Pyridyl imidazolidinone	VP1	C2	1.552 μM	>50 μM	VP1: V192M	*In vitro*[45], [47]
	BPROZ-103	Pyridyl imidazolidinone	VP1	C2	0.130 μM	>50 μM	VP1: V192M	*In vitro*[45], [47]
	BPROZ-299	Pyridyl imidazolidinone	VP1	C2	0.021 μM	>50 μM	VP1: V192M	*In vitro*[47]
	BPROZ-101	Pyridyl imidazolidinone	VP1	A, B1, C2	0.001 μM	>50 μM		*In vitro*[46], [47]
	BPROZ-160	Pyridyl imidazolidinone	VP1	C2	0.011 μM	40.1 μM	VP1: V192M	*In vitro*[47]
	BPROZ-161	Pyridyl imidazolidinone	VP1	C2	0.045 μM	28.3 μM		*In vitro*[47]
	BPROZ-033	Pyridyl imidazolidinone	VP1	A, B1, C2	0.008 μM	>50 μM	VP1: V192M	*In vitro*[47], [48], [221]
	BPROZ-074	Pyridyl imidazolidinone	VP1	A, B1, C2	0.0008 μM	>50 μM	VP1: V192M	*In vitro*[47]
	TJAB1099	Pyridyl imidazolidinone	VP1	C4	0.000025 μM			*In vitro*[49], [50]
	MADAL-385, Compound 22, Compound 30	Tryptophan dendrimers	VP1	A, B2, B5, C2, C4	0.0002–0.285 0.038–0.353 0.002–0.175 μM	24.9 μM >75.3 μM >100 μM		*In vitro*[54], [222], [223]
	AL-463	Tryptophan dendrimers	VP1	A	0.4 μM	>30 μM		*In vitro*[57]
	Compound IIa	Tryptophan dendrimers	VP1	A, B2	0.0017–0.20 μM	>19 μM		*In vitro*[56]
	AL-518	Tryptophan dendrimers	VP1	A	0.04 μM	>100 μM		*In vitro*[55]
	Suramin		VP1	A, B3, C4	40 µM	>100 μM		*In vitro*[18], [60] *In vivo*: mice and adult rhesus monkeys[61] Clinical: NCT03804749
	NF449 (Suramin analogue)		VP1 (5-fold axis of capsid)	A, B1	6.7 µM	130–1000 μM	VP1: E98Q and K244R	*In vitro*[59]
	Lactoferrin	Glycoprotein	VP1, downregulating IL-6, upregulating IFN-α	C2, B4	10.5–24.5 μg/mL (bovine) 103.3–185.0 μg/mL (human)	NR		*In vitro*[64], [65] *In vivo*: ICR mice[64] Clinical[66], [67]
	NiCS	Microcomposite	VP1	C2	300–500 μL (P < 0.01 by Student's paired two-tailed *t*-test)	>500 μL (3 g/mL)		*In vitro*[70]
	Brilliant black BN (E151)	Azo dyes	VP1 (5-fold axis of capsid)	A, B2, B4, B5, C1, C2, C4, C5	2.39–28.12 μM	1870 μM	VP1 E98K, G145E, P246A	*In vitro*, *In vivo*: AG129 mice[71]
	Glycyrrhiza uralensis	Herbal extract		A	0.056 μg/mL	>3000 μg/mL		*In vitro*[224]
	SMGGT	Herbal extract		A	0.21 μg/mL	>5000 μg/mL		*In vitro*[225]
	Artemisia capillaris	Herbal extract		A	90.8 μg/mL	>3000 μg/mL		*In vitro*[226]
	GLTA GLTB	Herbal extract			20 μg/mL (P < 0.01)	>100 μg/mL		*In vitro*[227]
Viral replication	3A12 2A10	mAb	RdRp	A, Mouse-adapted (MAV-VR)				*In vitro* *In vivo*: Neonatal ICR mice[74]
	Ribavirin	Nucleoside analogs		C2,	65 μg/mL (266 μM)	>200 μg/mL	RdRp L123F	*In vitro* *In vivo*: ICR mice[75], AG129 mice[76] Clinical: NCT02328651
	Gemcitabine	Nucleoside analogs		A	0.2–1 μM	>50 μM		*In vitro*[77]
	FNC	Nucleoside analogs	RdRp	C4	16.87 nM	3.238 μM		*In vitro* *In vivo*: Neonatal mice[78]
	NITD008	Nucleoside analogs	RdRp	A, B3, C	0.106–0.165 μM	>30 μM		*In vitro*[79]
	DTriP-22		RdRp	A, B4, C2	0.07–1.22 μM	>100 μM		*In vitro*[80]
	BPR-3P0128		RdRp, VPg uridylylation	C2	0.0029 µM	>20 μM		*In vitro*[81]
	2CL	Peptide	2C	C2, C4	1.35 μM	>150 μM		*In vivo*: ICR mice[87]
	12a, 19b, 19d	Fluoxetine analogue	2C	A, B5, C4	0.27–4.03 μM	>92.59 μM	M193 (19)	*In vitro*[89]
	6aw	Quinoline analogues	2C	C2	3.1–3.6 μM	32.4 μM		*In vitro*[91]
	6i	Quinoline analogues	2C	C4	1.238 μM	105.679 μM		*In vivo*: mice[92]
	JX001	Pyrazolpyridine compound	2C		2.3 μM	50 μM		*In vitro*[93]
	JX040	Pyrazolpyridine compound	2C		0.5 μM	>200 μM		*In vitro*[93]
	7d	Pyrazolpyridine compound	2C	C2	0.06–0.1 μM	>200 μM	2C-D183V 2C-D323G	*In vitro*[94]
	Itraconazole	Triazole	3A	C4	0.53–1.81 µM	>25 μM	3A: V51L, V75A	*In vitro*[97]
	Posaconazole	Triazole	3A	C4	1.29 μM	>10 μM		*In vitro*[97]
	GW5074	Enviroxime-like	3A	C4, B1	1.38–6.4 μM	96 μM		*In vitro*[97]
	AN-12-H5, AN-23-F6	Enviroxime-like	3A	B1	0.55 μM 0.15 μM	78 μM 84 μM	VP1: A224T VP3: R227K	*In vitro*[228]
	Camptothecin (C9911)	Alkaloid	TOP1	C2, B4		>10 μM		*In vitro*[98]
Viral protein translation	Idarubicin (IDR)	Anthracycline	IRES	A	0.493 μM	192.1 μM		*In vitro*[102]
	Daunorubicin (DNR)	Anthracycline	IRES	A	1.132 μM	>255.9 μM		*In vitro*[102]
	Epirubicin (*EPI*)	Anthracycline	IRES	A	1.415 μM	>75.07 μM		*In vitro*[102]
	Apigenin	Flavonoids	IRES	C4	10.3 μM	79.0 μM		*In vitro*[103], [104]
	Kaempferol	Flavonoids	IRES	C4				*In vitro*[103], [106]
	DMA-135	SM	IRES	C2	7.54 μM	>100 μM		*In vitro*[105]
	Quinacrine		IRES	C4	9.71 µM	>20 μM		*In vitro*[105]
	Amantadine		IRES	pGS-EV-A71				*In vitro*[108]
	vivo-MO-1	vivo-MOs	IRES	B4	1.5 µM	ND	VP2: T533C	*In vitro*[109]
	vivo-MO-2	vivo-MOs	IRES	B4, A,	1.2 µM	ND		*In vitro*[109]
	Curcumin	Natural phenolic compound	PKCδ	C2	10 µM (P < 0.01 by Student’s two-tailed *t*-test)	>40 μM		*In vitro*[109]
	miR2911	miRNA	VP1	C4				*In vitro*[111]
	Se@PEI@siRNA	siRNA	VP1	C4				*In vitro*[112], [115]
Viral protein processing	Rupintrivir (AG7088)	Peptide	3C	A, C2	0.018–0.8 µM	134 µM		*In vitro*[115] *In vitro*[44] *In vivo*: ICR mice[116]
	SG85	Peptide	3C	A	0.18 µM	265 µM		*In vitro*[121]
	NK-1.8 k	Peptidyl aldehyde	3C	A, B3, C4	0.09–0.105 µM	>200 µM		*In vitro*[116]
	NK-1.9 k	Cyanohydrin derivatives	3C	C4	0.037 µM			*In vitro*[126]
	Aldehyde 5X	Peptidomimetic Aldehydes	3C	EV-A71 (FY)-Luc	0.11 µM	>100 µM		*In vitro*[129]
	Compound 18P	Peptidomimetic Aldehydes	3C	A	0.03 µM	>100 µM		*In vitro*[120]
	DC07090		3C		22.09 µM	>200 µM		*In vitro*[134]
	Luteoloside	Flavonoids	3C	C4	430 µM	2300 µM		*In vitro*[135]
	Cyanohydrin (R)-1	Peptidomimetic compound	3C		0.048 µM	>100 µM		*In vitro*[131] *In vivo*: mice[132]
	FOPMC FIOMC	Peptidomimetic compound	3C	A, B, C4	0.115–0.135 0.075–0.102 µM	>200 µM		*In vitro*[133] *In vivo*: mice[132]
	Compound 2 K	Michael acceptor	3C	C4	0.028 µM			*In vitro*, *In vivo*: mice[124]
	SLQ-4 SLQ-5	Michael acceptor	3C	A, B3, C4	0.038–0.119 µM 0.093–0.239 µM	ND or >400 µM		*In vitro*[125]
	Chlorogenic acid (CHA)	Herbal extract	2A	A	6.3 µg/mL	121.5 µg/mL		*In vitro*[142]
	Rosmarinic acid (RA)	Herbal extract	2A, IRES	A, B4, C2	43.07 µg/mL	209.8 µg/mL		*In vitro* *In vivo*: ICR (suckling mice)[145]
	Schizonepeta tenuifolia Briq	Herbal extract	2A	A, B4, C2	1250 µg/mL (P < 0.01 by Student’s *t*-test)	3903 µg/mL		*In vitro*[144]
	LVLQTM	Peptide	2A	C4	200 µM			*In vitro*[143]
	2-hydroxymyristic acid	Myristic acid analogues	VP4	B4	250 µM	>500 µM		*In vitro*[26]
	4-oxatetradecanoic acid	Myristic acid analogues	VP4	B4	50 µM	>100 µM		*In vitro*[26]
	12-methoxydodecanoic acid	Myristic acid analogues	VP4	B4	250 µM	>500 µM		*In vitro*[26]
Progeny virus release	Retro-2^cycl^		vesicle transport	C4	12.56 µM	>500 μM		*In vitro* *In vivo*: Newborn BALB/c mice[149]
	Retro-2.1		vesicle transport	C4	0.05 µM	>267.80 μM		*In vitro* *In vivo*: Newborn BALB/c mice[149]

## Structure, typing, and life cycle of EV-A71

**Fig. 1:**
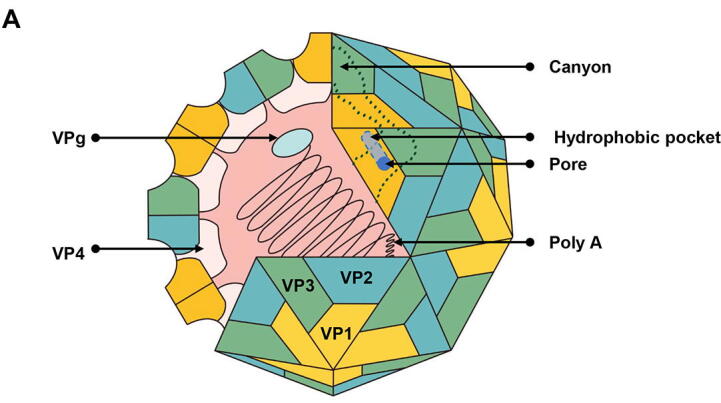

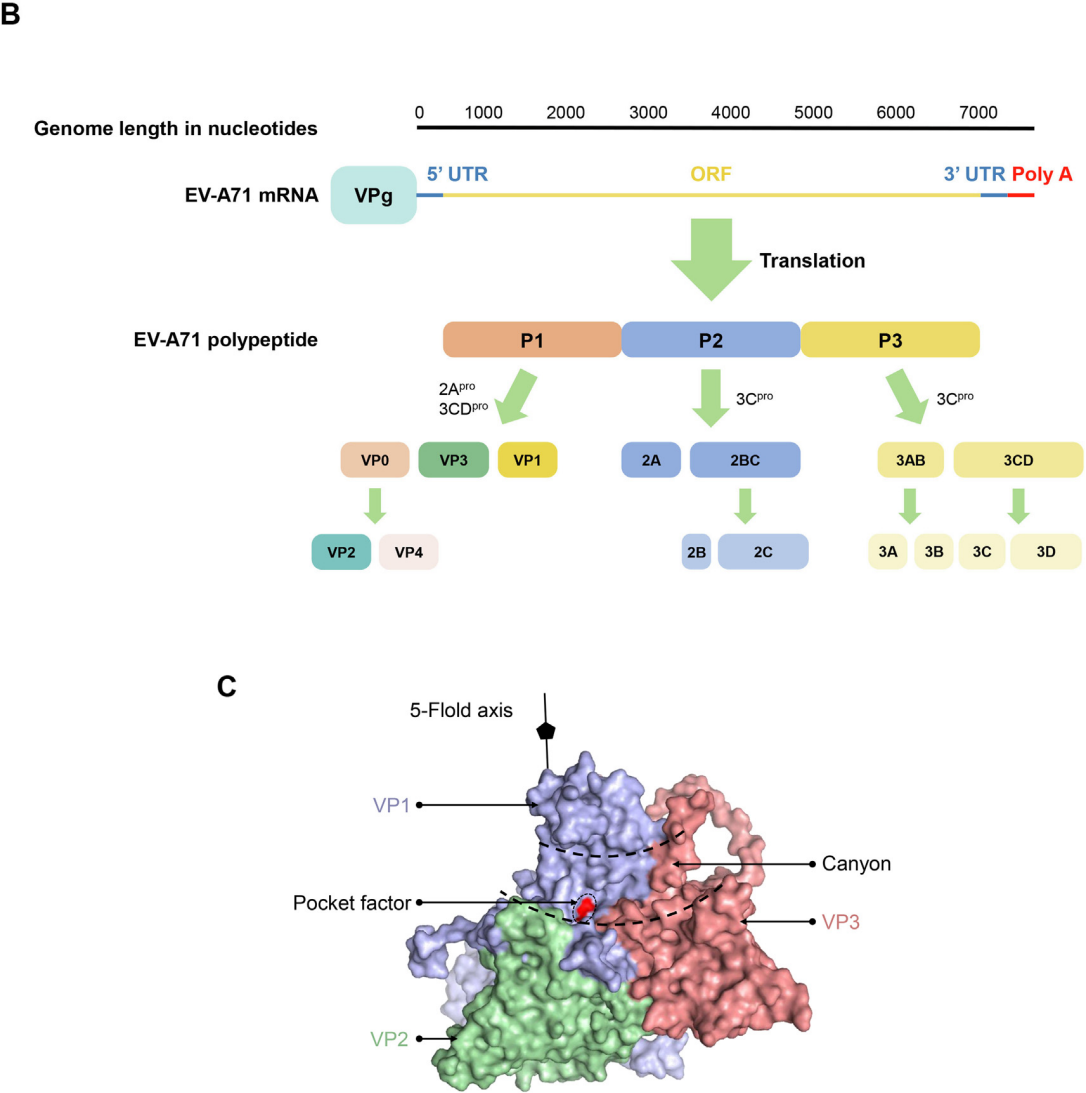
**The structure of EnterovirusA71. (A) The icosahedral particle structure of EV-A71.** EV-A71 is a non-enveloped, single-stranded, positive-sense RNA virus with an icosahedral structure. EV-A71 contains a polyadenylate (Poly A) tail at the end of the 3′-untranslated region (UTR) and a viral protein genome-linked (VPg) covalently bound to the 5′-UTR. The viral capsid protein consists of VP1, VP2, VP3, and VP4, where VP1, VP2, and VP3 become the outer surface of the viral particle, while VP4 is arranged inside the viral capsid to be the inner surface. VP1 and VP3 form canyons, the VP1 surface also includes hydrophobic pockets and pore. **(B) The genome structure of EnterovirusA71.** The EV-A71 virus genome is approximately 7.4 kb in length and contains only one open reading frame (ORF). The ORF is translated into polypeptides containing P1, P2, and P3. The P1 protein is cleaved by the virus-encoded 2A^pro^ and 3CD^pro^ to produce VP0, VP1, and VP3, where VP0 is the precursor protein of VP2 and VP4, ultimately becoming four structural proteins. The P2 and P3 proteins are cleaved by the virus-encoded 3C^pro^ to produce seven non-structural proteins (2A, 2B, 2C, 3A, 3B, 3C, and 3D). **(C) Viral capsid structure and location of canyon.** Surface rendering of one icosahedral asymmetric unit (PDB code: 3VBS) of EV-A71. EV-A71 capsid protein VP1, VP2 and VP3 are colored in light blue, pale green and salmon, respectively. A 5-fold axis is shown as a black line and the pocket factor (red) indicated by a black arrow.

EV-A71 is a non-enveloped virus with a single-stranded RNA genome of positive polarity (+) RNA. It is a member of the family *Picornaviridae* of small enteroviruses, whose viral particles have an icosahedral structure with a diameter of approximately 30 nm (Fig. 1A) [8]. The total length of the EV-A71 genome is approximately 7.4 kb, with about 10% of the non-coding region at each end. The integrity of the 3′ -terminal polyadenylate tail determines the infectivity of the virus, whereas the 5′ -terminal polyadenylate covalently binds to a small molecule protein (VPg) associated with virus replication [8] (Fig. 1B). The open reading frame encodes a polyprotein consisting of 2193 amino acids that can be cleaved by proteases into three precursor proteins P1, P2, and P3 [9]. P1 can be further cleaved into four structural proteins that constitute subunits of the viral capsid, of which VP4 is located in the interior, and the three surface proteins VP1-3 form an eight-stranded anti-parallel β-barrel. In most enteroviruses, the 5-fold axis of capsid symmetry is surrounded by a “canyon”, at the end of which the hydrophobic pocket amino acids on VP1 are highly conserved, but the internal pocket factor (possibly a lipped, fsphingosine or fatty acid) is specific and is partially exposed in EV-A71 [10], [11], [12]. (Fig. 1C) While P2 and P3 are cleaved into various non-structural proteins, such as EV-A71 autologous protein lyase and RNA-dependent RNA polymerase [13]. EV-A71 is divided into three genotypes, A, B, and C, according to the VP1 sequences on the surface of the virus capsid. B and C each contain five subtypes, B1–5 and C1–5, among which the B5 strain has caused outbreaks in Asian countries, while the C4a subtype is mainly responsible for the pandemic in mainland China [14].

**Fig. 2:**
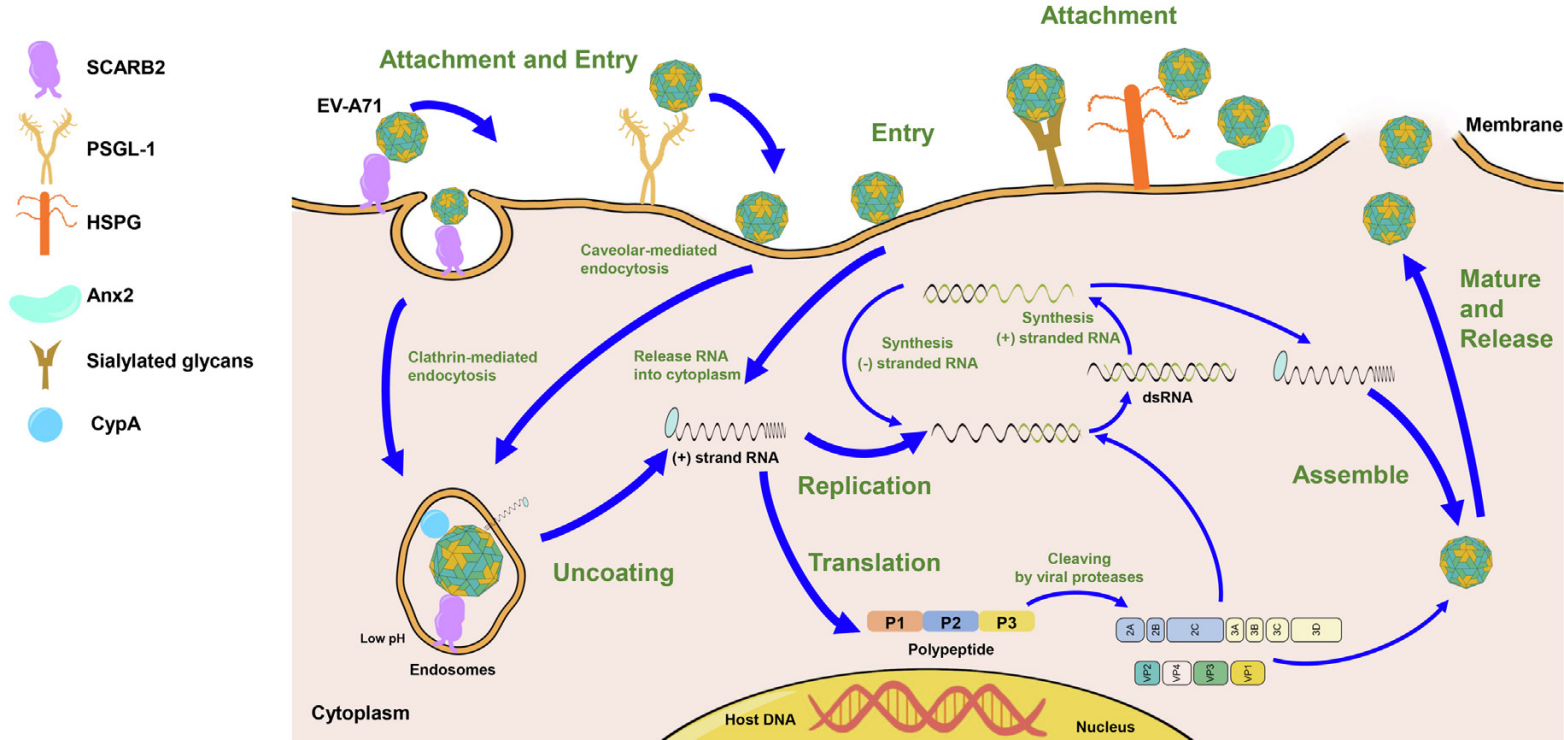
**The life cycle of EnterovirusA71.** EV-A71 binds to cellular receptors and is enriched on the cell surface. Anx2, HSPG, and sialylated glycans are only attachment receptors. In contrast, the EV-A71-SCARB2 complex enters the cell via endosomes by clathrin-mediated endocytosis. EV-A71 binds to PSGL-1 and enters the cell via endosomes by caveolar-mediated endocytosis. The endosomes with low pH and CypA or SCARB2 could mediate EV-A71 uncoating, and mRNA enter cytoplasm through the endosomal membrane via a small channels. In addition, by binding to specific receptors on the cell surface, the EV-A71 capsid protein causes structural change, and pores are formed on the cell membrane, allowing direct entry of the viral genome into the cytoplasm. After translation of the viral genomic RNA into a polypeptide, it is cleaved into 11 proteins by the virus-encoded proteases. The non-structural proteins are involved in the replication of the viral genome where negative-stranded RNA is synthesized and used as a template to synthesize the new positive-standard RNA. Nascent positive-stranded RNA could be used as a template to enter a new round of replication, or it could be assembled with a capsid formed by structural proteins to become a nascent virus particle, which is then released from the cell after forming a mature virus with infectivity by genomic regulation. Abbreviations: SCARB2: Scavenger Receptor Class B2; PSGL-1: P-selectin glycoprotein ligand-1; Anx2: Annexin Ⅱ; HSPG: heparan sulfate glycosaminoglycan; CypA: Cyclophilin A; (-) stranded RNA: negative-stranded RNA; dsRNA: double-standard RNA; (+) stranded RNA: positive-standard RNA.

Similar to most viruses, EV-A71 undergoes a routine life cycle of attachment, entry, uncoating, replication, translation, assembly and release (Fig. 2). Scavenger Receptor Class B Member 2 (SCARB2) [15], P-selectin glycoprotein ligand-1 (PSGL-1) [16], Annexin Ⅱ (Anx2) [17], heparan sulfate glycosaminoglycan (HS) [18], and sialylated glycans [19] on the cell surface are all EV-A71 attachment receptors that are capable of enriching the virus on the cell surface and enhancing viral infectivity [20]. Among them, SCARB2 is also the entry and uncoating receptor of EV-A71, and the structure of the EV-A71-SCARB2 complex has been reported in the literature using cryo-electron microscopy at 3.4 Å resolution (PDB: 6I2K). The complex formed by the binding of EV-A71 and SCARB2 can enter cells and accumulate in endosomes along the clahtrin-mediated endocytosis [21], [22], SCARB2 undergoes a conformational change at lower pH (≤5.6). As the receptor binding at the canyon, the pocket factor that previously stabilized the capsid passes through this tunnel to the endosomes membrane, during this process, the conformation of EV-A71 capsid is changed, and VP1 and VP4 form a channel in the membrane [11], to facilitate the release of the EV-A71 genome into the cell [11], [21]. In addition, EV-A71 can enter cells via caveolar-mediated endocytosis after binding to PSGL-1, but PSGL-1 cannot mediate EV-A71 uncoating. In endosomes under low pH conditions, Cyclophilin A (CypA) or SCARB2 mediates EV-A71 uncoating [23]. After EV-A71 binds to the specific receptor on the cell surface, the capsid protein undergoes a conformational change, and pores form on the cell membrane, allowing the viral genome to enter the cytoplasm directly [24]. However, the mechanism by which EV-A71 nucleic acids directly enter cells requires further study.

After releasing into the cytoplasm, viral genomic RNA is directly translated into peptides containing 2193 amino acids (P1, P2, and P3) under the action of host cell ribosomes. Subsequently, 2A proteinase (2A^pro^) catalyzes the cleavage at the N-terminal end of this polypeptide to produce the P1 protein. 3CD^pro^ cleaves P1 protein into VP1, VP3, and VP0, where VP0 is the precursor to VP2 and VP4, resulting in the formation of four structural proteins [25]. The myristylation signal of enteroviral VP4 is a highly conserved feature of enterovirus and a potential antiviral target [26]. 3C proteinase (3C^pro^) cleaves P2 and P3 into seven non-structural proteins (2A, 2B, 2C, 3A, 3B, 3C, and 3D), which are potential targets for antiviral drug development [8]. 3D^pol^, a viral RNA-dependent RNA polymerase (RdRp), participates in the replication of viral RNA and forms the RNA chain of progeny viruses [24]. The internal ribosome entry site (IRES) then interacts with the far-upstream element binding protein 2 (FBP2) and negatively regulates viral translation [27]. Finally, positive-stranded viral RNA is assembled with non-structural proteins and viral capsid proteins to form progeny infectious virus particles, which are released by the cell lysis pathway, thus enabling progeny viruses to enter a new life cycle.

## Viral-targeted drugs

### Inhibiting virus attachment and entry

The capsid protein of EV-A71 is critical for viral attachment and entry. Therefore, various drugs or capsid protein inhibitors that disrupt the interaction between viral capsid protein and receptor may be a potential treatment for inhibiting early EV-A71 infection.

#### Receptor

It has been reported that SCARB2 and PSGL-1 are the entry receptors of EV-A71 [15], [16].

Yamayoshi et al. demonstrated for the first time that SCARB2 is a receptor for EV-A71 by overexpressing human SCARB2 in L929, which is an EV-A71-insensitive cell line, enabling the production of progeny virus and cytopathic effects. In addition, soluble SCARB2 can competitively bind to EV-A71 with human SCARB2, indicating its potential as a drug candidate for EV-A71 [15]. In recent years, monoclonal antibodies have emerged as an ideal treatment for EV-A71 infections. One such antibody is JL2, which can compete with EV-A71 to bind to the extracellular region of human SCARB2（PDB: 5XBM）, thereby effectively inhibiting EV-A71 infection in target cells [28]. Two antibodies, A9 (PDB: 5ZUF) and D6 (PDB: 5ZUD), developed by Wang et al. showed effective neutralization activity by blocking EV-A71 binding to the receptor (neut_50_ of A9 and D6 were 0.1 nM and 1 nM, respectively), while the greater neutralizing potency of A9 may be due to its role in destabilizing the viral capsid [29]. In addition, chemosynthetic EV-A71 VP1 polypeptides have been shown to prevent viral attachment and to effectively reduce CPE induced by three EV-A71 strains (A, B, and C genotypes). Further studies revealed that the positively charged amino acids on the SP40 peptide are essential for its antiviral activity [30]. Feng et al. found that acarbose possesses the ability to bind EV-A71 competitively with SCARB2 in the living image system *in vivo*, which can block the dynamic transfer of viruses from the intestinal tract to the whole body. This suggests that such glycosyl compounds have the potential to act as viral entry blockers, but their universality needs to be validated by more experimental and clinical data [31].

PSGL-1 has also attracted attention as another receptor for EV-A71. Studies have shown that treatment with PSGL-1 antibody or interference with PSGL-1 expression can reduce MDDC-mediated EV-A71 transmission and rescue virus-induced cell death [32].

It has been reported that heparin sulfate on the cell surface is the adhesion receptor of EV-A71, and enriches the virus, enhances virus infection, and interacts with the entry receptor to promote virus entry into the cell [18]. Several investigators have demonstrated that heparin, heparan sulfate, and their analogs, as decoy receptors can exert anti-EV-A71 effects *in vitro* and their cytotoxicity is generally low [33], [34], [35]. Moreover, heparin reduces alterations in gene expression mediated by viral infection [35]. Monoclonal antibodies D5 (complex of EV-A71 and D5 fragment PDB: 3JAU) and C4 can simultaneously interfere with EV-A71 binding to SCARB2, PSGL-1, and heparin sulfate, which are expected to be highly effective blockers of viral attachment and internalization, promoting the development of monoclonal antibody-based treatment of EV-A71 infection [36].

In addition, EV-A71 also interacts directly with nucleolin on the surface of host cells via the capsid protein VP1. The use of anti-nucleoprotein antibodies can reduce the binding of EV-A71 to host cells. The reduction in EV-A71 binding after the knockout of nucleolin on the cell surface further demonstrates its potential as a drug target for mediating viral entry into cells [37].

#### Viral capsid

**Fig. 3:**
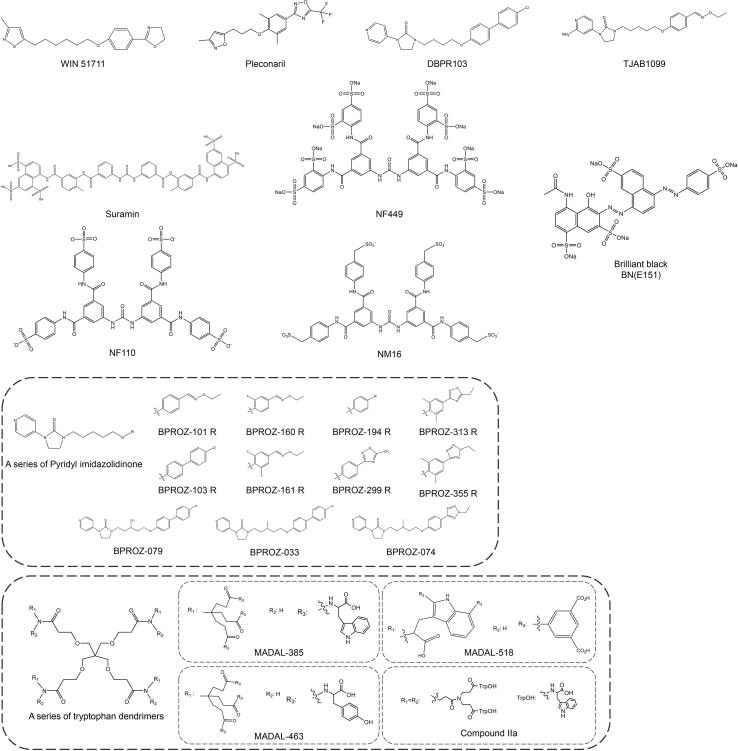
Chemical structures of EV-A71 capsid inhibitors.

The binding of receptor to the canyon region on the capsid triggers the uncoating process of EV-A71. It is actually due to the release of lipids from the hydrophobic pocket on VP1 that causes rearrangements of the capsid structure [38]. Huang et al. analyzed 500 enteroviral sequences [39] and 1632 VP1 sequences of the enterovirus [40], and identified the presence of conserved residues in the canyon region and hydrophobic pocket structure of VP1, indicating that the hydrophobic pocket of enterovirus is a conserved antiviral drug target. Naturally, researchers came up with the idea of stabilizing the capsid and preventing the release of the viral genome by replacing the pocket factor with a compound with a higher binding affinity to the conserved hydrophobic pocket [38]. WIN series of compounds is a kind of capsid binder that targets EV-A71 VP1 protein. Plevka et al. resolved the interaction mode of WIN 51711 (Fig. 3) and other compounds with the hydrophobic pocket by X-ray diffraction (PDB: 3ZFE) [38]. Pleconaril (Fig. 3), a broad-spectrum drug against picornaviruses, is effective in improving the survival of EV-A71-infected cells (EC_50_ on RD was 0.13–0.54 µg/mL) [10], [41], [42]. Simultaneously, pleconaril also has a beneficial effect in reducing early morbidity and mortality in EV-A71-infected mice [43], and could inhibit EV-D68 at the cellular level, but can’t inhibit Rhinovirus (HRV) 87 [44]. Unfortunately, just as pleconaril could not inhibit EV-A71 Taiwan isolates (1998), mutations located on VP1 may make EV-A71 resistant to the inhibitory effects of pyridyl imidazolidinones [45]. A series of pyridine imidazolidinones was synthesized using pleconaril and other WIN compounds as a matrix for modification and optimization, and their inhibitory effect on EV-A71 was investigated. For example, the SAR results of Chern et al. showed that the alkyl substituents on the oxime ether group and phenoxy ring largely influenced the *in vitro* anti-EV-A71 (three genotypes) activity of these new potent antiviral drugs, in which the ethyl oxime ether BPROZ-101 (Fig. 3) had an IC_50_ = 0.001 µM on RD cells without significant cytotoxicity [46], [47]. Except for EV-A71, BPROZ-101 also had inhibitory effects on four serotypes of Coxsackievirus A group (A9, A10, A16, A24), three serotypes of Coxsackievirus B group (B1, B4, and B5) and two serotypes of Echovirus (Echovirus 9 and 29). But there was no significant inhibitory effect on EV-D68 and CV-B2, B3 and B6 [46]. Chang et al. introduced methyl group at the 2- or 3- position of the linker between imidazolidinone and biphenyl on the basis of DBPR103 (Fig. 3), which improved the antiviral activity by more than four times [48]. Ma et al. also developed a gel disinfectant using the novel pyridyl imidazolidone, TJAB1099 (Fig. 3), as a formulation component, providing a potential means to prevent the public transmission of EV-A71 [49], [50]. Investigating the link between mutations and the antiviral effects of such compounds, Chen and Shih et al. found that Tyr (Y) 116 and Val (V) 192 of VP1 are key sites for pyridine imidazolidone (BPROZ-033/074/101/103/160/161/194/299) compounds (Fig. 3) to exert an antiviral effects [47], [51]. Currently, among such capsid inhibitors bound to hydrophobic pockets, NLD-22 (PDB: 6LQD) exhibits low toxicity and relatively the better antiviral activity *in vitro* (EC_50_ = 5.056 nM). The positive protective effect and pharmacokinetic profile of NLD-22 in animal studies make its subsequent development promising [52].

Li et al. screened a series of quinoline analogs with potential anti-EV-D68 based on structural optimization and structure-activity relationship evaluation, and identified a lead compound 19 that binds to the hydrophobic pocket of EV-D68 VP1, and the compound 19 has acceptable bioavailability in rats and significant metabolic stability in human liver microsomes [53], while the binding site of compound 19 (VP1 hydrophobic pocket) is conserved in enteroviruses [40]. Therefore it could further test the inhibitory effect of compound 19 to EV-A71 *in vivo* and *in vitro*, with potential for broad-spectrum antiviral.

In the last few years, HIV entry inhibitors with several tryptophan residues on their surfaces have emerged to inhibit the unrelated enterovirus EV-A71 (Fig. 3). Among them, the Cryo-EM structures of MADL385 (PDB: 6UH7) and CB-30 suggest that they inhibit the early stage of virus infection by targeting residues of EV-A71 structural protein VP1, especially the region forming the 5-fold axis of the viral capsid [54], [55], [56], [57].

The centuries-old drug suramin and its analogues (NF449, NF110 and NM16) (Fig. 3) have been reported to inhibit enterovirus A species [18], [58], [59]. Further studies have revealed that suramin blocks the attachment of EV-A71 to host cells by binding to a negatively charged naphthalenetrisulfonic acid group to a positively charged region around the 5-fold axis of the viral capsid, making it resistant to all single-charged alanine mutants [60]. Suramin with nontoxic concentrations can save mice and adult rhesus monkeys infected with a lethal dose of EV-A71 and has been identified as a clinical candidate for the treatment of EV-A71 infection (NCT03804749) [61]. Suramin could inhibit various of enterovirus, including CV-A2, A3, A9, A10, A12, A16, two EV-A71 strains and Echovirus 20, 25. But suramin had no inhibitory effect on CV-B3, B4, poliovirus (PV) 1, 2, 3 and EV-D68 [62].

Lactoferrin is one of the main protein components of breast milk and has a wide range of anticancer, antibacterial, antiviral, antifungal and antiparasitic activities [63]. By binding to VP1 of EV-A71, lactoferrin plays a role in the viral attachment stage while being able to induce the expression of interferon α (IFN-α) and down-regulate the expression of interleukin-6 (IL-6) induced by EV-A71 infection. Bovine and human lactoferrin has been shown to exhibit *in vitro* activity against EV-A71 with mean IC_50_ values of 10.5–24.5 μg/mL and 103.3–185.0 μg/mL, respectively [64], [65]. The survival rate of infected pups that ingested recombinant porcine lactoferritin milk was significantly improved, suggesting the potential of oral administration of lactoferrin-rich milk to prevent EV-A71 infection, but there was no supporting data in a 2002–2003 clinical trial of lactoferritin formulations [66], [67]. Therefore, the clinical efficacy of lactoferrin in the prevention or treatment of EV-A71 infection remains unclear. In addition, lactoferrin could inhibit PV [68] and Echovirus infection [69].

Nickel ion/chitosan (NIC) microcomposite and the azo dye brilliant black BN (E151) (Fig. 3), were also found to inhibit EV-A71 entry. The binding of nickel ion to VP1 is critical for NIC microcomposite to prevent EV-A71 from entering cells and uncoating [70], [71]. The safety of E151 as an edible dye speaks for itself, with an EC_50_ of 2.39–28.12 µM for various EV-A71 strains, such as B2, B5, C1, and C4. Furthermore, E151 elutes the attached virus and inhibits the interaction between EV-A71 and the decapacitating factor, CypA [71].

### Inhibiting viral replication

#### 3D polymerase

Like other *Picornaviridae*, the 5′ end of the positive strand of EV-A71 RNA is covalently linked to the VPg during replication [13]. VPg is uridylated under the action of 3D^pol^ (RdRp, which is a unique and conserved enzyme required for viral genome replication [72], [73]) and becomes a protein primer for RNA synthesis [13]. The negative and positive strands of viral RNA are then further synthesized alternately using RdRp. Therefore, drugs targeting RdRp similar to monoclonal antibodies against EV-A71 3D^pol^ can inhibit EV-A71 replication *in vitro* and *in vivo*, making them candidates for treatment [74]. In view of the importance of VPg site activity on enterovirus replication, drugs that can inhibit VPg activity have the potential of broad-spectrum anti-enteroviruses.

**Fig. 4:**
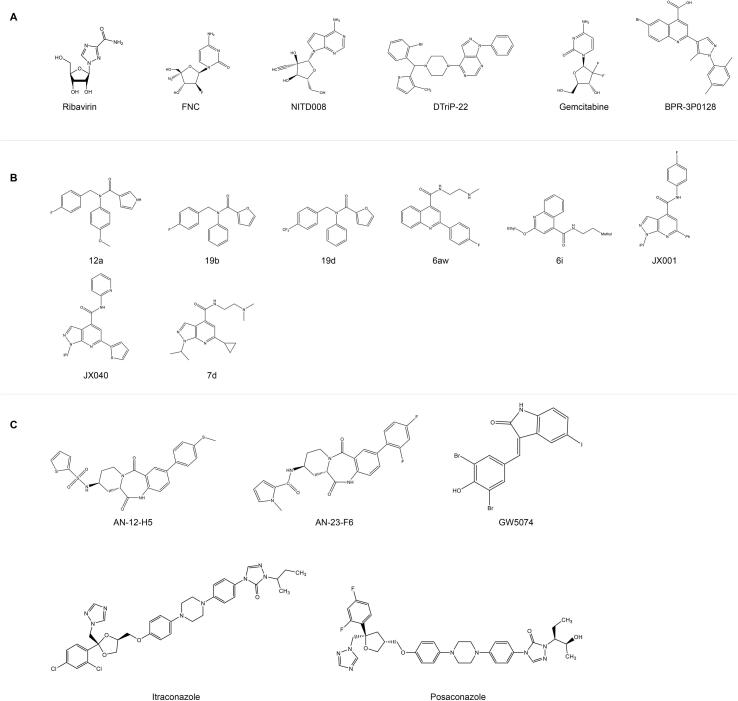
**Chemical structures of EV**-A**71 replication-related inhibitors.** (A) Chemical structures of EV-A71 3D inhibitors. (B) Chemical structures of EV-A71 2C inhibitors. (C) Chemical structures of EV-A71 2A inhibitors.

Ribavirin is often used as a positive control during EV-A71 drug development, with an IC_50_ of 65 µg/mL (266 µM) on RD cells (Fig. 4A) [75]. It is a nucleoside analogue that inserts into the viral genome during viral RNA replication and further reduces viral load by inducing and accumulating lethal mutations [75]. Meng et al. obtained the EV-A71 mutant RdRp-L123F with high fidelity and low pathogenicity compared with the wild-type EV-A71 B5 through a continuous passage in the presence of ribavirin, providing a new idea for the development of live attenuated and inactivated EV-A71 vaccines [75], [76]. The synergistic antiviral effect of ribavirin in combination with other drugs such as gemcitabine (Fig. 4A) has made it a common clinical treatment nowadays [77]. Other nucleoside analogues, such as FNC (Fig. 4A) [78], NITD008 and its triphosphorylated products (Fig. 4A) [79], interrupt viral RNA replication chains by binding directly to EV-A71 RdRp and both are more than ten times as effective as Ribavirin at the cellular level (EC_50_ for comparison). Nucleoside analogs FNC can widely inhibit a variety of enteroviruses, such as CV-A6, CV-A16, EV-D68, CV-B3 [78].

The non-nucleoside analogue DTriP-22 also showed inhibitory activity against EV-A71 RdRp *in vitro* polymerase analysis (Fig. 4A) [80]. DTriP-22 showed broad-spectrum inhibitory effects on CV-A9, A10, A16, A24, B1, B2, B3, B4, B5, B6, Echovirus 9, and HRV-2 [80]. Velu et al. used DTriP-22 as a control and verified that BPR-3P0128 (EC_50_ = 0.0029 µM) has the same antiviral mechanism but the better antiviral effect, which could be attributed to its further inhibition of VPg uridylation activity [80], [81]. However, BPR-3P0128 has not been reported against other enteroviruses (Fig. 4A).

#### 2C p*rotein*

The nonstructural protein 2C has been widely reported to have ATPase activity and helicase activity, which plays an irreplaceable role in viral replication. In addition, 2C also helps EV-A71 escape from TNF-α-mediated NF-κB immune response by inhibiting IKKα/β phosphorylation [82], [83], [84], [85]. As the most conserved protein in picornavirus, 2C protein has become a hot target for some broad-spectrum antiviral inhibitors in recent years.

Based on the crystal structure of the soluble part of EV-A71 2C (PDB: 5GRB), Guan et al. demonstrated that 2C carboxyl-terminal mediated self-oligomerization (generally hexamers) is the basis for 2C ATPase activity (positions 116–329), providing insights into structur-based drug design [86]. 2CL and B-2CL are structurally designed peptides based on EV-A 2C and EV-B 2C, respectively, and they could effectively attenuate the oligomerization of 2C protein, and 2CL could effectively protect EV-A71-infected mice [87].

Fluoxetine is a broad-spectrum antiviral targeting 2C, but it aggravates the symptoms of patients infected with EV-D68, indicating that safer and more effective small molecules should be developed [88]. Bauer et al. used fluoxetine analogs as parental modifications, and eventually identified three compounds 12a, 19b and 19d (Fig. 4B) were able to inhibit all tested EVs (such as EV-A71, CV-B3, PV-1, CV-A24, and EV-D68) and HRVs (such as HRV-A2 and HRV-B14). Subsequent studies with drug-resistant mutants revealed that the α2 helix of 2C is a conserved binding pocket for these inhibitors [89].

Similarly, Dibucaine, a local anesthetic targeting 2C, exhibited potent antiviral activity against enterovirus. Initially, Musharrafieh et al. chemically modified dibucaine to obtain a series of quinoline derivatives that optimized its efficacy against EV-D68 [90]. Subsequently, a stepwise optimization strategy was adopted to modify quinoline analogues, and identified 6aw (Fig. 4B) with broad-spectrum antiviral activity against EV-D68, EV-A71 and CV-B3 and long half-life in mouse microsomes [91]. Meanwhile, other researchers found that a low-toxicity derivative of dibucaine, 6i (Fig. 4B), can be administered orally (6 mg/kg) to protect EV-A71-infected mice, and the combination of compound 6i and emetine (an IRES inhibitor, 0.1 mg/kg) can completely prevent and treat the symptoms of mice infected with EV-A71 [92].

Pyrazolopyridine core in the molecular structure is a common feature of many anti-enterovirus drugs. In the early stage, Xing et al. modified a series of pyrazolopyridine compounds by combining conformational relationships with JX001 as a precursor, among which the 2-pyridyl amide JX040 significantly reduced the EC_50_ of EV-A71 and CV-B3 inhibition with low toxicity, but its anti-poliovirus effect was reduced (Fig. 4B) [93]. Later, compound 7d (4- positively charged amine with a hydrophobic alkyl linker) stood out with a selection index of over 2000 and focused on two resistance mutation sites, 2C-D183V and 2C-D323G, by resistance selection (Fig. 4B) [94]. There are a lack of *in vivo* animal model studies for these compounds, and further optimization based on *in vivo* efficacy or toxicity may be required in the future.

On the other hand, targeting the 2C-binding proteins TRIM4, exportin2 and ARFGAP1 could indirectly inhibit the normal function of viral 2C and may serve as a new antiviral therapeutic route [95].

Except for 3D and 2C, the non-structural proteins 3A (PDB: 6HLW) or 3AB are involved in the formation of replication complexes, which in turn participate in the early life cycle of the virus [96]. The antifungal agent itraconazole and the cycloxime compound AN-12-H5, can inhibit EV-A71 replication by targeting the 3A protein in the low concentration range (EC_50_ is 1.15 µM and 0.55 µM, respectively) (Fig. 4C) [97]. In addition, DNA topoisomerase 1 (TOP1) plays a key role in both RNA replication and protein synthesis of EV-A71 virus, but removal of TOP1 from genome is not lethal, and camptothecin can be used as a TOP1 inhibitor against EV-A71 infection [98].

### Inhibiting viral protein translation

**Fig. 5:**
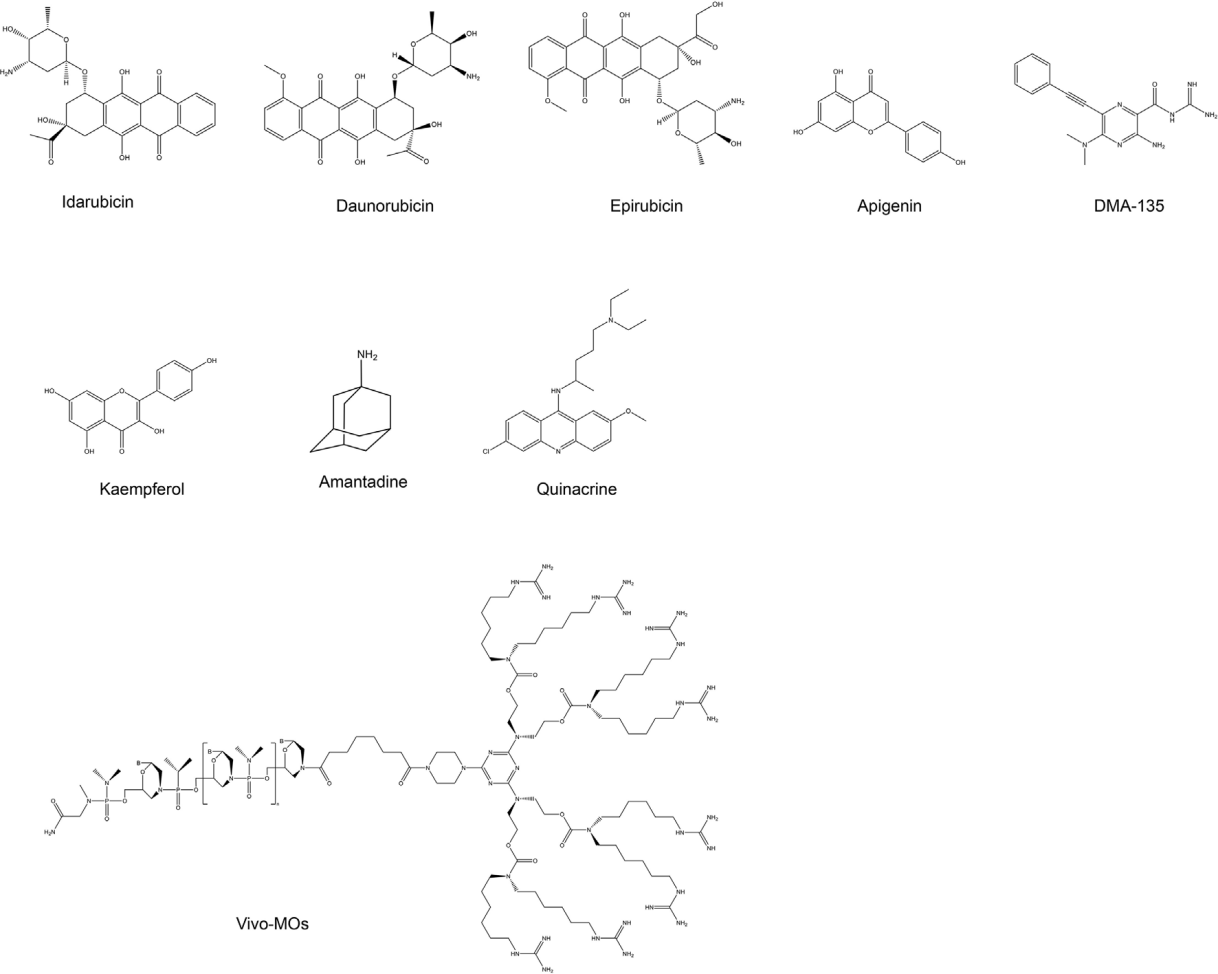
**Chemical structures of EV**-A**71 IRES inhibitors.**

The type I IRES of EV-A71 is located within the untranslated region at the 5′ end of the viral genome, which recruits intracellular ribosomes under the regulation of trans-acting factors (ITAF) such as nuclear proteins fructose-bisphosphatase 1 (FBP1) [99], fructose-bisphosphatase 2 (FBP2) [27] and heterogeneous nuclear ribonucleoprotein A1 (hnRNP A1) [100], [101], and drives viral protein translation initiation, is a conserved site for enteroviruses and even picornavirus. Anthracycline (such as Idarubicin (IDR), Daunorubicin (DNR), and Epirubicin (EPI)) [102] and apigenin [103], [104] affected the interaction between hnRNP A1 and EV-A71 IRES at the submicromolar and micromolar levels, respectively, and effectively inhibited viral translation (Fig. 5). Among them, anthracycline showed broad-spectrum antiviral activity against various enteroviruses, such as EV-A71, CV-A16, CV-B1, CV-B2, Echovirus 9, and Echovirus 30 [102]. AU-rich element binding factor 1 (AUF1) competitively inhibits hnRNP A binding to EV-A71 stem loop II (SLII) protrusions, thereby counteracting translation stimulation. As an allosteric inhibitor, DMA-135 (Fig. 5) effectively increases the affinity of AUF1 for the IRES SLII structural domain by binding to SLII, thus stabilizing the ternary complex AUF1-SLII (DMA-135) and achieving translation inhibition (PDB: 6XB7) [105]. FUBP and HNRP are also trans-acting factors that interact with EV-A71 IRES, and the natural herbal ingredient kaempferol prevents more than 80% of RD cells from cytopathy by interacting with both (Fig. 5) [106]. Similarly, quinacrine [107], amantadine [108], vivo-MO-1, and vivo-MO-2 [109] all inhibit a variety of enteroviruses, including EV-A71, by inhibiting IRES-mediated cap-independent translation (Fig. 5), vivo-MOs could inhibit PV, CV-A16 and EV-A71 [109]. Small molecule DMA-135 was speculated to have a broad-spectrum antiviral effect on EV-D68, but further verification is required [102].

In addition to directly affecting EV-A71 IRES activity, drugs can also affect viral translation by regulating host protease activity. For example, the anticancer drug curcumin inhibits EV-A71 replication in intestinal epithelial cells by reducing the phosphorylation of protein kinase Cδ (PKCδ), a key enzyme for viral translation [110]. With the development of gene editing technology in recent years, the direct targeting of viral genes by designing specific nucleotide sequences has provided new ideas for antiviral drug development. Li [111] and Lin [112] successively designed miRNA (miR2911) and siRNA to directly target VP1 for post-transcription gene expression regulation.

### Inhibiting viral protein processing

Polyproteins synthesised in ribosomes are cleaved by proteases to become functional viral proteins and precursor proteins, which is a key step in viral maturation. The 3C^pro^ and 2A^pro^ of EV-A71 play vital roles in this process.

#### 3C proteinase

In addition to participating in the processing of most protein precursors, 3C^pro^ also affects toll-like receptors (TLRs), RIG-I-like receptors, Nod-like receptors (NLR) family PYRIN domain containing-3 (NLRP3), IFN, and other related signalling pathways by hydrolysing host proteins, and plays an important role in inhibiting host innate immunity and triggering programmed cell death [113]. 3C^pro^ has no homology with mammalian proteases [113], and His40-Glu71-Cys147 catalytic triad have similar and stable conformations [114], which underlies its feasibility as an anti-EV-A71 target.

**Fig. 6:**
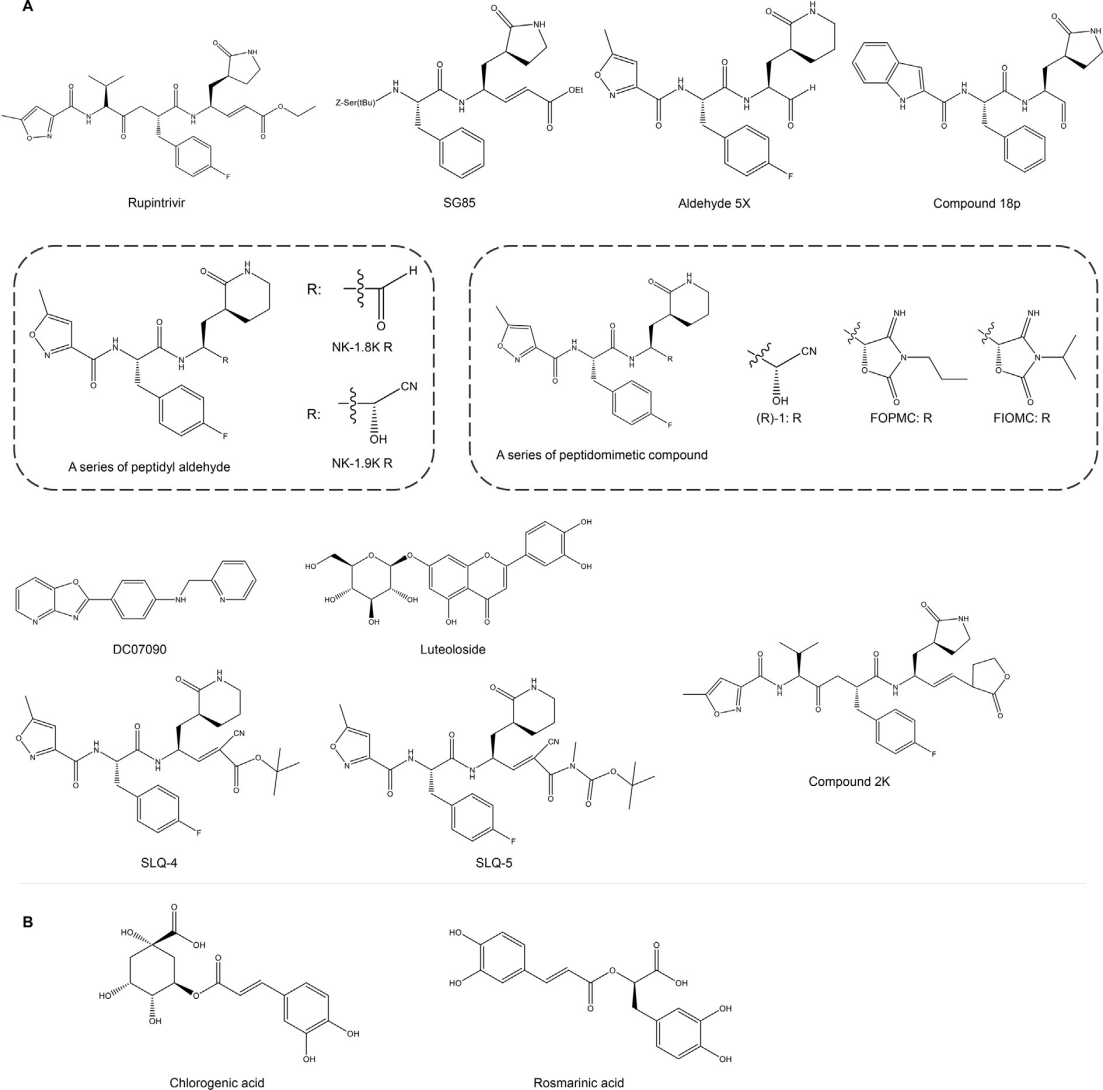
**Chemical structures of protein processing related inhibitors.** (A) Chemical structures of EV-A71 3C inhibitors. (B) Chemical structures of EV-A71 2A inhibitors.

Among the drugs targeting EV-A71 3C^pro^, peptidomimetic compounds are hot spots for the design and synthesis of efficient new drugs. Rupintrivir (AG7088) (Fig. 6A), a representative peptidyl compound, has been validated for its antiviral activity at molecular [115], cellular [44], and individual [116] levels, making it a promising candidate for the treatment of severe cases of EV-A71 infection [117]. Lu et al. speculated that the structural and chemical complementarity of rupintrivir and 3C is key to exerting antiviral effects. They provided ideas for structural modification of 3C inhibitors by revealing that the semi-closed S2 subunit and the reduced size S1′ subunit in EV-A71 3C protease are the limiting factors for binding to rupintrivir P1′ (PDB: 3SJO) [118]. The α, β-unsaturated alkyl ester on Rupintrivir is the Michael receptor for the cysteine residue and was first developed as an inhibitor of human rhinovirus 3C^pro^, but it is generally tolerated in early clinical trials [119]. Subsequently, Tan et al. combined the structure of EV68 3C^pro^ with Rupintrivir and designed a series of Michael receptors whose antiviral effect increased with the length of the unsaturated alkyl ester. Several peptidomimetic aldehydes such as the rupintrivir and its analogues could inhibit EV-D68, HRV 87 and CV-A21 [44], [120]. Among them, SG85 showed excellent activity against enteroviruses, including EV-A71, with an EC_50_ of 180 nM (Fig. 6A) [121], and could effectively and broadly inhibit nine strains of HRV-A (RV02, 09, 15, 29, 41, 59, 63, 85, 89), five strains of HRV-B (RV14, 42, 70, 72, 86), CV-A3 [122], CV-A16 [123], and EV-D68 [121]. In 2016, Li et al. also improved the pharmacokinetic stability of Rupintrivir in mouse plasma by introducing the lactone Michael receptor [124]. Recently, Liu et al. introduced cyano and carbonyl substituents at P1′ as double-activated Michael receptors to improve drug inhibition [125]. The development of peptidomimetic compounds has been ongoing, and Wang [126], [127], Ang [128], Zhai [129] and Dai [129] have designed and synthesized a series of 3C^pro^-specific inhibitors, among which aldehyde 5x has the most potent 3C^pro^ inhibitory activity (IC_50_ = 0.10 ± 0.02 μM), while NK-1.9 k (3C^pro^ and NK-1.9 k complex PDB: 5GSO) has the most prominent antiviral activity (EC_50_ = 24.9 nM), at the same time, NK-1.9 k also has the inhibitory effect on EV-D68 [126] (Fig. 6A). It was also found that the introduction of an aromatic ring at the R1 group, an aldehyde at the P1 position [120], and the presence of trifluoromethyl [130] enhanced the antiviral effect of peptidyl compounds. Surprisingly, compound 18p (3C^pro^ and 18p complex PDB: 7DNC) not only has stronger anti-enterovirus activity but also effectively inhibits SARS-CoV-2 compared to parent rupintrivir, while its good pharmacokinetics strongly promotes its development as a broad-spectrum antiviral drug (Fig. 6A) [120].

3C^pro^ is essentially a cysteine protease, and since 2015 cyanohydrin has gradually attracted attention as a functional group that selectively targets cysteine proteases to inhibit EV-A71 infection [131]. Zhai et al. found that the -CN group and electrophilicity of α-carbon play an important role in the binding of cyanohydrin to EV-A71 3C^pro^ and obtained the highly selective EV-A71 inhibitor cyanohydrin (R)-1 (EC_50_ = 0.048 ± 0.006 μM) by continuous targeted modification of cyanohydrin derivatives [131]. Based on cyanohydrin derivative (R)-1, the more stable isomers of cyanohydrin FOPMC and FIOMC [132], [133] were obtained by replacing the acyl cyanohydrin group with 4-iminooxazolidin-2-one, which provided a new idea for the original structural scaffold of EV-A71 drug development (Fig. 6A).

Since peptidomimetic drugs may be hydrolysed by the host of the virus, the small-molecule inhibitor DC07090 [134] and the natural extract luteoloside [135] have certain significance as non-peptidomimetic 3C inhibitors (Fig. 6A).

#### 2A proteinase

Non-structural protein 2A is involved in the hydrolysis of P1/P2 and 3C/3D, complementing the polyprotein processing of protease 3C [136], [137]. Wang et al. compared the 2A full-length sequences of representative enteroviral species (including enterovirus A-L and rhinovirus A-C), and found that Ser/Thr125 and His-Asp-Cys catalytic triad are highly conserved in enterovirus [138] and are important sites for the development of broad-spectrum antivirals. In addition, activator of transcription 2A, together with 3C^pro^, suppresses host immune activation affected by the antiviral IFN response by hydrolysing NLRP3 [139], [140]. The crystal structure of the EV-A71 2A protease C110A mutant (PDB: 4FVB) and its binding to the substrate complex (PDB: 4FVD) determined by Cai et al. using X-ray diffraction laid the foundation for the design and development of antiviral drugs against EV-A71 2A protease targets [141]. Chlorogenic acid at 20 µg/mL can block the transcription and translation of EV-A71 2A^pro^ in RD, thereby blocking protease 2A^pro^ at the source (Fig. 6B) [142]. LVLQTM peptide inhibits enzymatic cleavage of 2A^pro^ by competing for its active site and exerts antiviral effects *in vitro* and *in vivo* [143]. Chen et al. found that similar to LVLQTM peptides, extracts of *Schizonepeta tenuifolia* Briq and *Melissa officinalis* inhibit CAP-dependent translation initiation by interfering with 2A^pro^ hydrolysis to form eIF4G, while inhibiting processes such as hnRNP A1 transport and reactive oxygen species (ROS) -induced p38 kinase activation, resulting in multi-targeted inhibition of EV-A71 [144], [145]. Zhang et al. optimized the structure of 3-benzylcoumarins to inhibit the activity of EV-A71 2A^pro^ [146], and Musharrafieh et al. identified Telaprevir as an inhibitor of EV-D68 2A^pro^ [147]. However, there is a lack of studies on the inhibition of other enterovirus with current 2A inhibitors. Given it is highly conserved, 2A^pro^ has the potential to be an effective target for the broad-spectrum inhibitors.

### Inhibiting progeny virus release

As mentioned above, 3C^pro^ is highly correlated with apoptosis, and apoptosis induced by late infection facilitates the release of daughter viruses; therefore, targeting 3C^pro^ directly or targeting 3C^pro^ substrates such as the telomere-binding protein PinX1 may provide a strategy for drug development [148]. Retro-2^cycl^ and Retro-2.1 were first used as toxin inhibitors, and Dai et al. found that they inhibited EV-A71 to varying degrees *in vitro* and *in vivo* (Select index of 39.81 and 5356 in 293S cells, respectively) [149]. Moreover, the autophagy inhibitor, 3-MA, may block further viral infection by inhibiting the production and release of infectious EV-A71 particles triggered by cellular autophagy [150].

## Drugs targeting the host

### Regulating host immunity

EV-A71 infection triggers a series of immune responses (innate or adaptive) in the host to fight the pathogen, which is the reason that HFMD can heal spontaneously very quickly in most cases. However, it has been demonstrated that EV-A71 can escape the immune response by cleaving host proteins using proteases 2A^pro^ and 3C^pro^ to alter the IFNs expression pathway [151], [152]. In contrast, excessive and strong proinflammatory chemokine and cytokine responses in the host after EV-A71 infection are associated with disease severity [153]. Therefore, appropriate use of immunomodulators based on patient symptoms may be an effective adjunctive therapy to prevent EV-A71 infection.

EV-A71 infection has been reported to trigger the activation of TLRs, which can initiate MyD88-dependent or TRIF-dependent signalling cascades, leading to the activation of the nuclear factor kappa B (NF-κB) and mitogen-activated protein kinase (MAPK) pathways, inducing a proinflammatory cytokine storm [154]. Resveratrol [155] and the shikonin ester derivative PMM-034 [156] inhibited EV-A71 *in vitro* by blocking NF-κB p50/p65 phosphorylation. In addition, aqueous extract SCB [157], cathelicidin antimicrobial peptide LL-37 [158], and 9 k and 9 m [159] synthesized by Sun et al., were found to modulate host immune responses by inhibiting ERK phosphorylation, p38 MAPK phosphorylation and MEK1 allosteric activity, respectively.

The IFN response is an important defence mechanism in the antiviral immune response. Early studies have confirmed that treatment with Type I IFN at the initial stage could protect mice from EV-A71 infection, while treatment with IFN at the later stage of infection is virtually ineffective [160]. In contrast, Huang et al. obtained supportive data for IFN treatment in a 2016 clinical trial conducted in Henan, China. Recombinant human IFN α1b (rHuIFN-α1b) was administered by both ultrasonic aerosol inhalation and intramuscular injection in patients with HFMD. Treatment with rHuIFN-α1b could shorten the time of fever in patients, but the limitations in the sample scope make it necessary for further clinical validation of the safety and efficacy of this treatment modality [161]. In addition, human IFN-α/ω has shown positive effects *in vitro* experiments to prevent EV-A71 infection in RD, Caco-2, and SK-N-SH cell lines [160]. However, the protective effect of IFN-α on the host is still limited; for example, it cannot inhibit EV-A71 replication in the Vero cell line. Surprisingly, the combination of Rupintrivir (a kind of 3C^pro^ inhibitor) with IFN-α at a ratio of 1:40 or 1:200 has a synergistic anti-EV-A71 effect, with a combination index that reaches 0.14–0.27 [119].

In addition to direct administration of IFN therapy, aloe-emodin [162], all-trans-retinoic acid [163], natural saponin anisoside B4 [164], ginsenoside Rb1 [165], antimicrobial peptide LL-37 [158], and *Andrographis paniculata (Chuanxinlian)* and its derivatives [166] as IFN inducers were able to stimulate human cells to produce additional IFN-α, IFN-β, and IFN-γ to reduce the apoptosis of infected cells. As a targeted agonist of TLR7, both compound R837 [167] and GS-9620 [154] were able to activate the immune pathway, and prevent mice from limb paralysis and death after EV-A71 infection, although GS-9620 caused diarrhoea in mice. On the other hand, researchers have found that EV-A71 infection regulates IFN expression at the post-transcriptional level, and developed antiviral targets with relevant miRNAs targeting IFN. Available results show that EV-A71 infection upregulates miR-548 and miR-146a, which inhibit IFN-λ1 (type III IFNs) and IFN-β (Type Ⅰ IFNs) post-transcription, respectively. The corresponding miRNA inhibitors can restore IFN expression and prevent the host from being infected *in vitro* and *in vivo* [168], [169]. Contrarily, Li et al. found that miR-9 expression is downregulated after EV-A71 infection in Vero, RD, and HT-29 cell lines and mouse models. They further showed that overexpression of miR-9 downregulates NF-κB and proinflammatory cytokine expression, maintaining a moderate level of immunity to limit EV-A71 infection [153].

Intravenous immunoglobulin (IVIg), extracted from donor plasma and rich in neutralising antibodies, has also played an important role in treating infectious diseases caused by various viruses. Similarly, using IVIg in the clinical treatment of patients with severe diseases such as brainstem encephalitis caused by EV-A71 infection [170], [171] and pulmonary oedema [171] has received mostly beneficial feedback. However, because of the high specificity of IVIg, it tends to neutralise only specific pathogenic subgenotypes from the donor’s plasma or partially non-antigen-determining mutant subgenotypes, whereas it is ineffective for other “non-customized” subtypes [6], [172], [173]. Therefore, the combination of IVIg with drugs such as the immunomodulator milrinone may provide better supportive therapy for severe patients infected with EV-A71 [170], [174]. Similarly, hyperimmune plasma therapy is a form of passive immunisation. In small clinical trials, children with EV-A71 neurological complications were cured within three days without significant neurological sequelae [175].

### Inhibiting autophagy or apoptosis

Autophagy and apoptosis are interrelated and together determine cell fate [176]. Therefore, inhibiting autophagy or apoptosis induced by EV-A71 infection *in vitro* and *in vivo* during infection is an effective means to hinder viral replication and the pathogenesis of EV-A71-related diseases [177], [178].

EV-A71 induces autophagy in cells, and inhibition of autophagy during the autophagosome formation stage can indirectly inhibit virus release [177], [178]. It has been shown that mTOR and ERK are two key pathways closely associated with EV-A71-induced autophagy in mice [179]. The PI3K/Akt/mTOR signalling pathway, with mTOR as a key molecule, regulates cell growth and environmental responses. Rapamycin is a recognised autophagy inducer that specifically inhibits mTOR [180]. Torin2 is also an efficient mTOR inhibitor [180]; however its water solubility is poor. Hao et al. synthesized thirty derivatives based on Torin2, five of them showed similar mTOR kinase inhibitory activity, and 11e (IC_50_ = 0.027 µM) had the closest anti-EV-A71 effect to Torin2 *in vivo* (IC_50_ = 0.01 µM) [181]. Inhibition of JNK signaling pathway is another way to target autophagy induced by EV-A71 infection. JNK signaling pathway regulates autophagy mainly by phosphorylating Bcl2 family proteins and affecting their binding with Beclin-1 (a basic regulator of autophagy) [182]. Berberine and its derivatives play an antiviral role by downregulating the MEK/ERK signalling pathway in Vero cells. In addition, they inhibit JNK and PI3KIII phosphorylation-mediated autophagy [183], [184]. Wang et al. found that 4 µM of the lycorine derivative, LY-55, effectively reduced JNK phosphorylation. The reduction in the autophagy marker LC3Ⅱ and the increased expression of the selective autophagy receptor P62 in the presence of the drug demonstrated its potent autophagy inhibitory effect [185]. Surprisingly, the combination of LY-55 with the PI3K inhibitor 3-MA synergistically inhibited autophagy triggered by EV-A71 and CV-A16. The host restriction factor APOBEC3G (A3G) is effective against retroviruses, such as HIV-1, HBV, and HPV [186]. It has been identified that A3G affects viral translation and replication by competitively binding to the 5′-UTR of EV-A71. Unfortunately, the autophagy-lysosome pathway mediated by EV-A71 non-structural protein 2C induces A3G degradation, and thus overcomes A3G suppression [186], [187]. In contrast, some host factors, such as autophagy-related protein 4 homologue B (in RD cells) [188], m6A methyltransferase METTL3 (in Schwann cells) [189], and autophagy-promoting protein Beclin-1 [190], play an active role in autophagy and apoptosis induced by EV-A71. Targeting key host genes may provide new insights into drug development.

Apoptosis can be regarded as an antiviral response of the body to maintain the homeostasis of the internal environment after viral infection. On the other hand, programmed cell death and inflammatory factor storm induced by EV-A71 infection are considered to be the cause of severe disease and death [191].

EV-A71 induces caspase-dependent apoptosis, in which caspase-3 is an important component of the cytotoxic T cell killing machinery. Caspase-3 shears the poly ADP-ribose polymerase (PARP) after activation by 3C^pro^, causing loss of custody of gene integrity [192]. *Grifola frondosa* polysaccharide [193] and extracts of *Houttuynia cordata* Thunb [194]. effectively inhibited the hydrolytic enzymatic activity of caspase-3 at 250 µg/mL and 125.92 ± 27.84 µg/mL, respectively, which led to a reduction in cytopathic effects. Moreover, 300 µg/mL of Gan-Lu-Siao-Du-yin effectively inhibited EV-A71-induced apoptosis by directly inhibiting caspase-8, an apoptosis initiator that triggers the caspase cascade, and indirectly inhibiting Bax [195]. Meanwhile, serine/threonine protein kinase Akt activated under the regulation of PI3K can also inhibit apoptosis by targeting NF-κB or phosphorylating BAD in various ways [154], [196]. Salvianolic acid B (a major component of *Salvia miltiorrhiza* root) [197], PML (polysaccharide from *Monostroma latissimum*) [198], MWS (a sulfated glucuronorhamnan from the green seaweed *Monostroma nitidum*) [199], and PPE (polysaccharides extracted from *Picochlorum* sp. 122) [200], inhibit apoptosis by activating of the Akt/PI3K signalling pathway. In recent years, research led by Wang [201] and Cao [202] prepared functionalized anti-EV-A71 nanoparticles RES-NPs (200 g/mL resveratrol loaded onto nanoparticles) and SeNPs@OT (20 nM oseltamivir loaded onto surfaces of 9.8 μM selenium nanoparticles) to prevent autophagy or apoptosis in infected cells, and provided a reference for further improvement of drug utilisation.

### Regulating the cellular redox environment

Oxidative stress is a key factor in neurological diseases caused by viral infections [203]. EV-A71 infection downregulates the peroxisomal protein acyl-CoA oxidase 1, which in turn triggers excessive ROS accumulation, destroys the normal intracellular redox environmental balance, and finally leads to apoptosis and autophagy in neuronal cells, such as SK-N-SH and U251 [204]. Both apigenin and luteolin (one hydroxyl group difference in the B-ring) can effectively inhibit the production of ROS and the expression of inflammatory factors, such as IL-6, in the host with minimal cytotoxicity. However, unlike luteolin, apigenin can also directly target and inhibit EV-A71 IRES to limit viral replication via multiple targets [205]. The cytoprotective protein deglycase (DJ-1)/nuclear factor erythroid 2-reated factor 2 (NRF2)/haem oxygenase 1 (HO-1) pathway (DJ-1/NRF2/HO-1) is an intracellular antioxidant pathway [204], and overexpression of HO-1 and the presence of its metabolite carbon monoxide inhibit the formation of ROS and the replication of EV-A71 to protect SK-N-SH cells from EV-A71 infection-induced death [203]. Ho et al. found that 50 µM of polyphenolic compounds epigallocatechin gallate and gallocatechin gallate significantly inhibited EV-A71 replication in oxidative damage-sensitive cells (glucose-6-phosphate dehydrogenase (G6PD)-deficient HFF1 cells) [206]. In addition to G6PD, glutathione reductase and glutathione peroxidase are also involved in regulating intracellular redox homeostasis, and isochlorogenic acid C (ICAC) treatment could modulate the above antioxidant enzymes and restore the ROS content and GSH/GSSG ratio to normal levels in infected cells. Furthermore, the survival rate of EV-A71-infected mice injected intraperitoneally with 6.4 mg/kg ICAC was higher than that of mice injected with 10 mg/kg Ribavirin, with survival rates of 60% and 50%, respectively [207].

### Other host factors

In addition to the host factors mentioned above, CypA is a pH-dependent regulator of viral uncoating, but the VP1-S243P mutation confers resistance to CypA [208]. Yan et al. chemically modified two small molecules of CypA inhibitors. The final derivative, compound 11 (EC_50_ = 0.37 µM, CC_50_ > 25 μM), had excellent synergistic antiviral effects in the higher concentrations range (0.31–5 μM) in combination with the 3C^pro^ inhibitor NK-1.8 K [209].

## Conclusion

HFMD is a global infectious disease that poses a public health challenge. Therefore, vaccines and personal hygiene measures are necessary to prevent HFMD. In addition, ultraviolet light, ozone, heat, and chlorine-containing disinfectants play important roles in inactivating the virus and blocking EV-A71 *in vitro* transmission [210], [211], [212], [213], [214]. However, specific drugs against EV-A71 remain unavailable. Ribavirin (nebulised inhalation/oral) [215] is currently used clinically to treat mild HFMD caused by EV-A71, but owing to the safety and adverse reactions of Ribavirin, the WHO and FDA have strictly restricted the use of Ribavirin in paediatric patients (https://www.who.int/publications/i/item/WHO-MHP-HPS-EML-2021.03). Like other viral diseases, recombinant human IFN still has great potential for short-term treatment of HFMD; however more attention should be paid to the dose, duration, and range of treatment to minimise adverse effects such as fever and pain. In addition, there are clinical treatments that use antiviral drugs, such as acyclovir, dexamethasone, and sulforaphane, as adjunctive therapy, but their effectiveness or safety remains controversial. In immunocompromised children and severely ill patients, in addition to the use of common symptomatic drugs, it has been reported to be treated with intravenous IVIG or plasma exchange treatment, combined with adjuvant means such as mechanical ventilation if necessary [151], [216]. In conclusion, despite significant advances in EV-A71 drug development within the last 20 years, there is still a lack of safe and effective EV-A71 inhibitors at the clinical stage.

This article reviews potential drugs that target various stages of the EV-A71 life cycle and play a role in host regulation to provide a basis for further EV-A71 drug development (Table 1). Researchers have identified hundreds of EV-A71 inhibitors based on screening repurposed drugs, targeted structural design, and rational modification of previously effective drugs as the main development strategies. Enterovirus capsid inhibitors appeared as early as the last century and developed at the fastest rate, but the narrow antiviral spectrum became its shortcoming. Among them, lactoferrin has the advantage of high efficiency and non-toxicity, whereas pyridyl imidazolidinone compounds have become the most effective candidates (EC_50_ up to pM) for targeting the virus by solving the resistance problem of some strains through continuous modification and optimisation. As the structure of the EV-A71 viral protein continues to be resolved, structurally diverse inhibitors targeting viral IRES/3D/2C/3C/2A have come into view [3], [8]. Although their *in vitro* inhibition effect was overall inferior to that of the capsid inhibitors, few compounds were eliminated after resistance selection assays [89]. Currently, there are relatively less drugs targeting structural protein VP4, 3A and other non-structural proteins, which warrants further development. In contrast, new antivirals targeting essential cellular factors may have the advantage of being broad-spectrum and less susceptible to be drug resistant [217]. Until specific drugs are available, combination therapy with drugs targeting different targets or immunomodulators may be an effective and universal treatment for continuously evolving EV-A71 variants [92], [119]. The fact that most drugs are in preliminary development suggests that the development of *in vivo* trials should be accelerated to assess their clinical efficacy in a timely manner.

Traditional drug development is based on the discovery of new targets based on the resolution of viral protein structures by X-ray diffraction, nuclear magnetic resonance and cryoelectron microscopy, followed by the targeted design or modification of molecular structures to obtain potential antiviral compounds [11], [105], [141]. The potential for moving the drug candidates to human clinical trials was further evaluated through drug resistance screening, and drug half-life and efficacy evaluation *in vivo*. The maturation of infectious cloning techniques and the construction of intestinal organoid platforms in recent years have accelerated the process of drug evaluation [12]. At the same time, drug analogs with high affinity for viral proteins can be used as fluorescent probes for tracking and visualizing the studied enteroviruses by combining with various tags, facilitating the further exploration of the viral life cycle and transport pathways *in vivo* [218].

In conclusion, current limited knowledge on the virus and its mutants hinder the development of potent drugs. Accelerating related research and developinging effective broad-spectrum anti-enterovirus drugs will play an invaluable role in the treatment of acute viral infection, and eventually reducing retaed mortality and alleviating the epidemic. In addition, the application of CRISPR, RNAi, TALEN and other gene editing tools to screen for host factors affecting EV-A71 infestation is important to further explore the mystery of the EV-A71 life cycle. With the understanding of the molecular characteristics and replication processes of EV-A71 and other enteroviruses, the development of new potent therapeutic and safe drugs is within reach.

## Funding

This study was supported by the National Natural Science Foundation of China (Grant No. 82151224, No. 82202492), 10.13039/501100012166National Key Research and Development Program of China (Grant No. 2020YFA0712102, BWS21J025, and 20SWAQK22), Key Project of Beijing University of Chemical Technology (Grant No. XK1803-06, XK2020-02), Fundamental Research Funds for Central Universities (Grant No. BUCTZY2022), and H&H Global Research and Technology Center (Grant No. H2021028).

## Declaration of Competing Interest

The authors declare that they have no known competing financial interests or personal relationships that could have appeared to influence the work reported in this paper.
